# Perception of Substance Use Disorder Training: a Survey of General Psychiatry Residents in Nigeria

**DOI:** 10.1007/s40596-021-01433-y

**Published:** 2021-03-24

**Authors:** Eze U. Chikezie, Ikenna D. Ebuenyi, Erefagha Leonardo P. Allagoa, Ifeoma N. Onyeka

**Affiliations:** 1 Niger Delta University Teaching Hospital, Amassoma, Bayelsa Nigeria; 2 Maynooth University, Maynooth, Ireland; 3 National Postgraduate Medical College of Nigeria and Federal Medical Centre, Yenagoa, Bayelsa Nigeria; 4 Queen’s University Belfast, Belfast, UK

**Keywords:** Psychiatry residents, Substance use disorders, Addiction training, Satisfaction, Addiction psychiatry

## Abstract

**Objective:**

Substance use disorder (SUD) is a global concern. Evidence from high-income countries suggests that SUD training for psychiatry residents is less than optimal but it is unknown whether the situation is different in low-/middle-income settings. This study assessed psychiatry residents’ perception of their SUD training.

**Methods:**

A cross-sectional survey was conducted among general psychiatry residents in Nigeria from November 2018 to May 2019. Data were collected through self-completion of an English-language questionnaire with multiple-choice and open-ended questions administered face-to-face and online.

**Results:**

A total of 51 participants completed the questionnaire, mean age 33.6 years and 76.5% men. Most participants (70.6%) expressed interest in addiction psychiatry, and 47.1% perceived their SUD training as inadequate. When asked to rate satisfaction with the SUD training they have received so far, 52.9% were unsatisfied, and the absence of in-house SUD training (29.4%) was the leading cause of dissatisfaction. For those who were satisfied, the most common reasons were availability of SUD training and treatment-related factors (31.4%). The most frequent suggestions for making addiction psychiatry subspecialty attractive to psychiatry residents were provision of SUD treatment units, structured SUD training, and continuity of such training. Equipping existing SUD treatment units and creating more treatment units were the most common suggestions for improving current SUD training.

**Conclusion:**

This study demonstrated a high level of interest in addiction psychiatry, but satisfaction with SUD training was mixed. Addressing causes of dissatisfaction and areas suggested for improvement would be necessary to sustain interest.

Substance use is prevalent globally. According to the 2019 World Drug Report [1], past-year users of any substance among those aged 15–64 years increased globally from 210 million in 2009 to 271 million in 2017. The rise was mainly driven by updated substance use data from some countries including Nigeria. Results from the first large-scale nationwide substance use survey in Nigeria revealed that 14.4% or 14.3 million people aged 15–64 years had used substances (excluding alcohol and tobacco) at least once in the past year in 2017 [2]. Prevalence by substance type was 10.7% for cannabis, 4.7% for opioids (mainly non-medical use of prescription opioids such as tramadol, codeine, or morphine), 2.4% for cough syrups containing codeine and dextromethorphan and under 1% for cocaine, amphetamines, ecstasy, hallucinogens, and solvents/inhalants [2]. In Nigeria, treatments for SUDs are mainly available in tertiary hospitals, but limited services are also available through faith-based and non-governmental organizations [2]. Some treatment centers were designed primarily to treat SUDs, while others treat SUDs as a secondary problem within the context of psychiatric treatment [3]. However, only 12% of high-risk substance users in Nigeria had ever received treatment [2], and only 1 in 7 persons with SUDs receive treatment each year globally [1].

Psychiatrists play very prominent roles in treating mental health disorders, including SUDs [4]. There have been concerns about SUD training for general psychiatry residents, including unavailability of addiction training in psychiatry residency programs and problems with the nature of the training ranging from curriculum content and structure, duration of clinical experience, quality of supervision, and timing of the training [5–7]. Limited diversity in the training sites where psychiatry residents see SUD patients is a notable concern. Seeing SUD patients in emergency rooms or inpatient units may perpetuate negative perceptions of SUD patients as being difficult and poorly responsive to treatment with high attrition rates [7, 8]. Residents also need exposure to SUD patients in outpatient settings who are less impaired and successfully recovering from addiction [5, 6]. A well-structured SUD training helps residents develop self-awareness of their biases and gain useful knowledge and skills that engender positive attitude toward patients with SUDs [9].

One question warranting attention is whether psychiatry residents favorably perceive the SUD training offered to them. Only a few studies have explored this topic. Khouzam [10] assessed the opinions of 24 psychiatry residents in the USA regarding their SUD rotation. One question asked, “How would you rate your satisfaction with the rotation?” A majority (95%) rated an above-average satisfaction with their rotation [10]. A review of reflective papers of 28 psychiatry residents in Canada reported that most were satisfied with their training, but the actual number or proportion of satisfied residents was not mentioned [9]. None of these studies explored reasons for satisfaction and dissatisfaction. Additionally, existing studies on SUD training for psychiatry residents and perceptions of SUD training were conducted in high-income countries. It is unknown whether the situation is different in low-/middle-income settings, which is important given the large psychiatric treatment gap in such countries [11] and the rating of SUDs as one of the disorders needing greater attention [4]. Given the limited resources in low-/middle-income settings, we hypothesized that SUD training may not be perceived favorably, and we developed this study to assess psychiatry residents’ perception of SUD training in Nigeria.

## Methods

We conducted a cross-sectional study among general psychiatry residents in the six regions (or geopolitical zones) in Nigeria from November 2018 to May 2019. We adopted a purposive sampling method, whereby all doctors training to specialize in psychiatry were eligible to participate in the study. In Nigeria, general psychiatry residency training consists of a minimum of  four years divided into two stages. The first stage is junior residency (i.e., pre part 1), which lasts a minimum of 24 months and qualifies psychiatry residents to sit for the part 1 fellowship examination. The second stage is senior residency (i.e., post part 1), which lasts another minimum of 24 months and qualifies psychiatry residents to apply for the final part 2 fellowship examination [12]. Psychiatry residents undertake SUD training during junior residency. If not available in-house, SUD training could be completed at an accredited external private or public institution. Data were collected from the participants through self-completion of questionnaires administered face-to-face during the Association of Psychiatrists in Nigeria (APN) annual conference held at Jos in 2018 and online via psychiatry residents’ social media groups using the electronic version of the questionnaire. We adopted this dual data collection method due to logistics and the financial complexities of surveying psychiatry residents in six regions of the country.

Data were collected using an English-language questionnaire consisting of 14 multiple-choice questions and six open-ended questions. The questionnaire collected sociodemographic details (without names or any personal identifiers), information about psychiatry residency training in general and specifically for SUD training. One of the questions asked participants to rate their satisfaction with the SUD training they have received so far (very unsatisfied, unsatisfied, satisfied, very satisfied) and one open-ended question each asked for reasons for satisfaction and dissatisfaction respectively. Open-ended questions were also included to elicit suggestions on how to make subspecialty training in addiction psychiatry more attractive to psychiatry residents and how to improve current training in treating people with SUD. A section of the questionnaire assessed participants’ preparedness to treat SUD using sub-questions adapted from the Addiction Training Scale [13]; in this particular paper, however, only data regarding SUD training were presented.

Statistical Package for Social Sciences (SPSS) software version 21 (IBM Corporation, Armonk, NY) was used for statistical analysis. Findings were presented as frequencies, proportions, mean, and standard deviation (SD). Responses to the open-ended questions were heterogeneous so the results were manually summarized and common themes were grouped together. Approval for this study was obtained from the research ethics committee of the Niger Delta University Teaching Hospital Okolobiri, approval number NDUTH/REC/0045/2017. Participants’ informed consent for face-to-face data collection was verbally solicited. For online data collection, accepting to proceed to the next page to complete the questionnaire signified consent.

## Results

A total of 51 psychiatry residents participated in the study. Using the 69 psychiatry residents in the directory of the National Association of Resident Doctors (NARD) as the denominator, the response rate in this survey was 74%. Table 1 shows the characteristics of the study population. The mean age was 33.6 years (SD=2.4 years, range: 27–41 years). Over three-quarters of participants (76.5%) were men and 68.6% were married. A quarter (25.5%) were from the southwest region of the country and the mean duration of residency was 2.1 years (SD=1.1, range: 1–5). A majority (92.2%) were at the pre part 1 (or junior residency) level. The majority of participants (80.2%) indicated an interest in specializing in a specific area of psychiatry, and addiction psychiatry was the predominant subspecialty of interest (29.4%), followed by child and adolescent psychiatry (17.6%) and general adult psychiatry (13.7%).

**Table 1:** Characteristics of general psychiatry residents and their perceptions of substance use disorder training (*N*=51)

Variable	*N*=51 *n* (%)
Age	
Mean (SD), years	33.6 (2.4)
Gender	
Male	39 (76.5)
Female	12 (23.5)
Interest in specific area of psychiatry	
Yes	41 (80.4)
No/undecided	10 (19.6)
Subspecialty of interest	
Child and adolescent psychiatry	9 (17.6)
Geriatric psychiatry	2 (3.9)
General adult psychiatry	7 (13.7)
Addiction psychiatry	15 (29.4)
Forensic psychiatry	6 (11.8)
Other	3 (5.9)
None/no response	9 (17.6)
Interest in addiction psychiatry	
Yes	36 (70.6)
No	11 (21.6)
Undecided	4 (7.8)
Adequacy of training in SUD management	
Yes	27 (52.9)
No	24 (47.1)
Satisfaction with the training received so far	
Very unsatisfied	0 (0)
Unsatisfied	27 (52.9)
Satisfied	23 (45.1)
Very satisfied	1 (2.0)
If satisfied, reason for satisfaction	
Adequate training and treatment-related factors	16 (31.4)
Good patient recovery after treatment	4 (7.8)
Wide variety of cases	4 (7.8)
Not applicable/no response	27 (52.9)
If unsatisfied, reason for dissatisfaction	
Lack of SUD treatment units	8 (15.7)
No in-house SUD training	15 (29.4)
Inadequate personnel (consultants, residents, other mental health personnel)	3 (5.9)
Not applicable/no response	25 (49.0)

*SUD*, substance use disorder; *SD*, standard deviation

Participants’ perception of their SUD training is shown in Table 1. Many of the psychiatry residents (70.6%) expressed interest in the field of addiction psychiatry, but nearly half (47.1%) reported their training in caring for patients with SUDs to be inadequate. Over half (52.9%) rated the SUD training they have received so far is unsatisfactory. Lack of in-house SUD training was the predominant cause of dissatisfaction (29.4%). Conversely, some residents rated their SUD training positively, and the most common reasons for satisfaction were SUD training and treatment-related factors (31.4%), specifically the presence of structured SUD training, adequate number of consultant psychiatrists, availability of SUD treatment units, and laboratory for toxicology screening. In the questions exploring reasons for their answers, the proportion of the “not applicable/no response” category was 52.9% (*n*=27) in the satisfied group and 49.0% (*n*=25) in the dissatisfied group.

Participants’ suggestions on how to make addiction psychiatry subspecialty more attractive to psychiatry residents and how to improve current SUD training are shown in Table 2. Provision of SUD treatment units (*n*=18), provision of structured SUD training (*n*=15), and continuous training (*n*=14) were the top three suggestions for making the addiction psychiatry subspecialty attractive. Most participants (*n*=17) suggested better-equipped treatment units and setting up more treatment units as measures for improving current SUD training. Other suggestions for improvement made by more than three respondents included provision of continuous assessment/evaluation, expanding SUD treatment options with more psychotherapy, employing more residents and consultant psychiatrists, and having more resource persons and better structured SUD training (Table 2).

**Table 2:** Suggestions for making addiction psychiatry subspecialty attractive and for improving current training

Suggestions	*N*=51 (*n*)*
**Making addiction psychiatry subspecialty attractive to psychiatry residents**	
Provide SUD treatment unit	18
Provide structured SUD training	15
Regular/continuous training	14
Increase the number of consultants	6
Employ more residents to reduce workload and stress	6
Provide good laboratory, diagnostic, and treatment equipment	5
Sponsorship to conferences and updates	3
Provide adequate/effective medications	3
Increase training duration	1
Provide good working conditions	1
Adequate mentoring by consultants	1
No response	3
**Improving current training in managing patients with SUD**	
Better-equipped treatment units and opening more treatment centers	17
Availability of resource persons	9
Continuous assessment and evaluation of psychiatry residents	8
Improved and better structured SUD training	7
Employing more consultants and residents	5
Providing wider/better treatment options with more of psychotherapy	4
Providing incentives and good working conditions to attract more doctors	3
Community-based rehabilitation	3
Flexible/versatile substance use disorder treatment	2
Government legislation on substances of abuse	2
Sponsorship to conferences	1
More funding for mental health	1
Introducing postgraduate diplomas, masters, and PhD programs	1
No response	1

*SUD*, substance use disorder

*Responses may exceed 51 due to multiple answers

## Discussion

In this survey of 51 psychiatry residents from various parts of Nigeria, a large proportion of the participants expressed interest in pursuing further specialization in addiction psychiatry. Almost half perceived their training on managing SUD to be inadequate. When asked to rate satisfaction with the SUD training they have received so far, over half of participants were unsatisfied, and the absence of in-house SUD training program was the leading cause of dissatisfaction. However, less than half were satisfied and the most common reasons for satisfaction were SUD training and treatment-related factors. The most frequent suggestions for making addiction psychiatry subspecialty attractive included the provision of SUD treatment units, structured SUD training, and continuity of such training. Equipping treatment units and creating more treatment units were the most common suggestions for improving current SUD training.

In this study, 70.6% expressed interest in the field of addiction psychiatry, which was higher than 44.5% reported in an American study [14]. At 47.1%, the proportion of residents satisfied with their SUD training was lower than 95% reported in a study of residents in the USA [10]. A Canadian study reported a high level of satisfaction with SUD training but data regarding the actual number or proportion of satisfied residents were not provided [9]. Neither of the American and Canadian studies explored dissatisfaction, and as such, we could not compare our results with theirs.

These findings have several implications for Nigeria. Issues related to SUD treatment units/centers were mentioned among the reasons for dissatisfaction with SUD training, and among suggestions for improving SUD training and for making addiction psychiatry attractive to general psychiatry residents. An earlier study [3] highlighted a shortage of and an uneven geographical distribution of SUD treatment centers in Nigeria. Therefore, provision of more SUD treatment centers and equipping existing centers should be a top priority. It is also important to consider SUD treatment units that adopt evidence-based practices within available resources, which would entail keeping abreast of and adopting what works best for various types of SUDs, embracing approaches that produce the most benefits to patients, training and retraining staff as needed, and monitoring and auditing practices [15].

Our findings also highlighted a need to review the structure of SUD training in Nigeria, because participants cited it as one of the ways to improve SUD training and to make addiction psychiatry attractive. Psychiatry residents undertake SUD training during junior residency, and it could be completed at an accredited external site [12]. In this study, absence of in-house SUD training was the predominant reason for dissatisfaction with SUD training; one possible explanation is that residents had to travel or relocate to the training sites. It would be necessary to evaluate resources available at those accredited external training sites to ensure uniformity and quality of training. Such evaluation should also seek to uncover if variations in experiences occur between those who completed their SUD training in-house versus external sites. Concerns about provision of psychotherapy and the availability of staff and resource persons should be addressed to improve training. Some participants suggested continuity of SUD training and continuous assessments/evaluations; online education platform is a cost-effective means of supplementing in-person training and for continuing medical education [16]. Additionally, online platforms should also be considered as flexible and efficient means for providing interventions, thereby improving access to SUD treatment [17].

Strengths and limitations of this study need to be acknowledged. Research studies relating to SUDs are predominantly conducted in high-income countries [18]. Most existing studies on SUD training were conducted in high-income countries, so this study provided insights from a low-/middle-income country. The study was representative, as the participants were drawn from the six regions in Nigeria. The sample size was small, however, which precluded subgroup analysis and limits generalizability of the results. Despite the limitations, this study provides useful information on SUD training and may stimulate further research on evaluating the process of educating psychiatric physicians to treat SUDs. Assessing psychiatry residents’ perceptions of SUD training would offer valuable feedback to program directors regarding the training itself and provide an opportunity to seek suggestions for improvement. It might be worthwhile to conduct multiple research surveys on the same cohort of psychiatry residents to gather feedback at various points during residency training because opinions and attitudes change over time [19]. In this study, the not applicable/no response category in satisfied and dissatisfied groups were likely to be respondents who skipped the question because they were dissatisfied and satisfied, respectively. It, however, highlighted a limitation of understanding why residents may be satisfied/dissatisfied with quality of SUD training and further supports collection of feedback at various points during residency training. Addressing the causes of dissatisfaction and areas suggested for improvement would require the buy-in and support of decision makers. A previous study [20] found that lack of time, lack of institutional support, lack of faculty expertise, and lack of training sites were barriers to SUD training. Therefore, future research should seek feedback from medical/residency directors to identify their opinions, needs, and challenges.
